# Plasma MIC-1 and PAPP-A Levels Are Decreased among Women Presenting to an Early Pregnancy Assessment Unit, Have Fetal Viability Confirmed but Later Miscarry

**DOI:** 10.1371/journal.pone.0072437

**Published:** 2013-09-12

**Authors:** Tu’uhevaha J. Kaitu’u-Lino, Katerina Bambang, Joseph Onwude, Richard Hiscock, Justin Konje, Stephen Tong

**Affiliations:** 1 Translational Obstetrics Group, Department of Obstetrics and Gynaecology, Mercy Hospital for Women, University of Melbourne, Heidelberg, Victoria, Australia; 2 Endocannabinoid Research Group, Department of Cancer Studies & Molecular Medicine, University of Leicester, Leicester, United Kingdom; 3 Ramsay Springfield Hospital, Chelmsford, United Kingdom; 4 Department of Anaesthesia, Mercy Hospital for Women, Heidelberg, Victoria, Australia; Chinese Academy of Sciences, China

## Abstract

**Background:**

We have recently shown first trimester Macrophage inhibitory cytokine-1 (MIC-1) and Pregnancy Associated Plasma Protein-A (PAPP-A) serum concentrations are depressed among asymptomatic women destined to miscarry. Here we examined whether plasma levels of MIC-1 and PAPP-A are depressed among women presenting to an Early Pregnancy Assessment Unit (EPAU), noted to have a confirmed viable fetus, but subsequently miscarry.

**Methods:**

We performed a prospective cohort study, recruiting 462 women in the first trimester presenting to EPAU and had fetal viability confirmed by ultrasound. We obtained plasma samples on the same day and measured MIC-1, PAPP-A and human chorionic gonadotrophin (hCG), grouping the cohort according to whether they later miscarried or not. To correct for changes in analyte levels across gestation, we expressed the data as Multiples of the normal Median (MoMs).

**Results:**

We recruited 462 participants presenting to EPAU at 5-12 weeks gestation. Most (80%) presented with symptoms of threatened miscarriage (e.g. abdominal pain, vaginal bleeding). 34 (7.4%) subsequently miscarried. Median plasma MIC-1 levels among those who miscarried were 50% of those with ongoing pregnancies (Miscarriage cohort MoM 0.50 (25^th^-75^th^ centiles: 0.29-1.33) vs ongoing pregnancies MoM 1.00 (0.65-1.38); p=0.0025). Median plasma PAPP-A MoMs among those who miscarried was 0.57 (0.00-1.12), significantly lower than those with ongoing pregnancies (MoMs 1.00 (0.59-1.59); p=0.036). Plasma hCG levels were also significantly depressed among those who miscarried compared to those with ongoing pregnancies. However, the performance of MIC-1 as a diagnostic marker to predict miscarriage in this cohort was modest, and not improved with the addition of hCG.

**Conclusion:**

MIC-1 and PAPP-A levels are significantly depressed in women presenting to EPAU with ultrasound evidence of fetal viability, but later miscarry. While they are unlikely to be useful as predictive biomarkers in this clinical setting, they probably play important roles in the maintenance of early pregnancy.

## Introduction

Miscarriage is the most common complication of pregnancy and occurs in 9-15% of clinically recognized pregnancies [1,2]. While approximately 50% are associated with chromosomal abnormalities [3], the remaining are euploid where miscarriage probably occurs as a result of suboptimal implantation. As such, a significant proportion of these euploid miscarriages are potentially salvageable.

A biomarker that can be applied when the fetus is still viable and predicts which pregnancies are likely to progress and which will miscarry may be useful for a number of reasons. Firstly, it could provide reassurance to highly anxious patients. Secondly, it could help clinicians stratify management and frequency of follow-up visits according to likely risk of miscarriage. Thirdly, such a predictive biomarker may have clinical utility to help target emerging therapies to those at high risk of miscarriage. Given the rapidly burgeoning literature that has improved our knowledge of early pregnancy, it is conceivable that treatments may be developed to arrest the pathological cascade leading to implantation failure, salvage pregnancies. For instance, pravastatin has been shown to rescue embryos in a miscarriage animal model [4]. A recent Cochrane review suggests progesterone administered to women with threatened miscarriage may be efficacious in decreasing the incidence of miscarriage [5]. While clinical trials of aspirin and heparin to prevent recurrent miscarriage have yielded mixed results and have not proven efficacious for sporadic miscarriage, newer generation anticoagulant agents merit evaluation [6].

Macrophage inhibitory cytokine 1 (MIC-1) is a member of the transforming growth factor-β superfamily. It localizes to the syncytiotrophoblast [7] and decidua [8], increases across the first trimester in serum [7] and is proposed to play an immunomodulatory role to facilitate pregnancy success [8]. PAPP-A is another placental protein that also may have roles favouring maintenance of pregnancy [9].

We have previously proposed first trimester serum MIC-1 [1] and PAPP-A [9] concentrations measured among asymptomatic women can predict subsequent miscarriage. While these initial reports utilized samples in a retrospectively collected biobank, we subsequently validated these findings in a large prospective cohort study [10]. Among 782 asymptomatic women destined to miscarry, first trimester serum MIC-1 levels were significantly decreased at 63% of normal levels (MOM 0.63, 25^th^-75^th^ percentiles 0.33-0.88), whereas PAPP-A were only 23% of normal values [10]. Importantly, at a fixed 10% false positive rate (90% specificity), a test combining MIC-1 and PAPP-A yielded 63% sensitivity and a 6.6 positive likelihood ratio in predicting miscarriage. While this predictive performance could be potentially further improved by the addition of other biomarkers, this already forms the platform of a promising biomarker test to predict miscarriage among asymptomatic women.

Given these promising results, we set out to assess the ability of plasma MIC-1 and PAPP-A concentrations to predict miscarriage in a different population. Early Pregnancy Assessment Units (EPAU) are clinical services managing patients in early pregnancy with potential complications. A common clinical presentation to EPAUs are women with symptoms of threatened miscarriage (vaginal bleeding ± pelvic pain).

We measured these analytes in a cohort of 462 women presenting to an EPAU in United Kingdom (most with symptoms of threatened miscarriage) where an intrauterine fetal viability was confirmed at the same presentation. We examined the ability of MIC-1 and PAPP-A to predict miscarriage in this cohort. Given hCG is used clinically as an early pregnancy biomarker, we also measured this analyte.

## Materials and Methods

### Participants

We performed a prospective cohort study, recruiting women presenting to The EPAU at Leicester Royal Infirmary in early pregnancy. Our EPAU reviews patients referred from General Practitioners or Midwives. The most common reason for presentation at EPAU are women with threatened miscarriage (vaginal bleeding ± abdominal pain). However, we are also referred patients for other reasons, for instance those who are anxious (e.g. prior miscarriages) or have had a previous history of ectopic pregnancy (where it is important to confirm whether the current pregnancy is located within the uterus).

Women were excluded if they had a history of illicit drug use, an ectopic pregnancy, any significant systemic illness such as diabetes or if they were heavy smokers (>20 cigarettes per day).

To be eligible for this study, there had to be confirmation of fetal viability at ultrasound on the day of recruitment (presence of fetal cardiac activity). At the same ultrasound, gestational age was determined by measuring the crown-rump length, referencing charts.

Baseline characteristics of all women were recorded including maternal age, Body mass index, ethnicity, parity and previous miscarriages (Table 1). Additionally, clinical outcomes were recorded, including whether a miscarriage had occurred or not (primary outcome). Miscarriage was defined as spontaneous loss of pregnancy at less than 20 weeks gestation. Obstetric details were also collected for those who had ongoing pregnancies, including delivery details.

**Table 1:** Characteristics of study participants.

	**Live births** (n=428)	**Miscarriage** (n=34)	**P**
**Age**	30.5 (0.3)	31.53 (1.2)	ns
**Body Mass Index**	25.7 (0.28)	26.8 (0.99)	ns
**Primiparous** -n (%)	183 (44)	12 (36)	ns
**Multiparous** -n (%)	244 (56)	21 (64)	ns
**Ethnicity** -n (%)			ns
**Caucasian**	331 (77.5%)	27 (81.8%)	
**Caucasian other**	18 (4.2%)	3 (9.1%)	
**Asian**	46 (10.8%)	-	
**Black**	12 (2.8%)	-	
**Mixed**	11 (2.6%)	-	
**Not recorded**	9 (2.1%)	3 (9.1%)	
**Number of previous miscarriages**	1.8 (0.13)	1.8 (0.36)	ns
**Gestation at blood sampling (weeks**)	7.8 (0.1)	6.9 (0.2)	0.0009
**Gestation at Delivery (weeks**)	39.4 (1.7)	n/a	n/a
**Birthweight (grams**)	3339 (29.8)	n/a	n/a

Data presented at Mean (±SEM) except where indicated. ns = not significnt. n/a = not applicable.

### Ethics statement

This study was approved by the Leicestershire and Rutland local research ethics committee. All women gave informed written consent to participate in the study.

### Sample collection

Approximately 9 mls of venous blood was collected into EDTA plasma tubes (Sarstedt, Leicester, UK). The tubes were centrifuged at 1200*g* at 4°C for 30 minutes, and the plasma collected and stored at -80°C until analysis. All samples were promptly processed and frozen within 60 minutes of sample collection by investigator K.B.

### Measurement of hCG, MIC-1 and PAPP-A

Samples were assayed for plasma levels of hCG (ALPCO, Salem, NH, USA), MIC-1 and PAPP-A (R&D Systems, Minneapolis, MN, USA) using commercially available ELISAs. Analytes were measured in plasma samples according to manufacturer’s instructions. The minimum detection limits for the respective assays were 5 mIU/ml for hCG, 7.81 pg/ml for MIC-1, and 0.78 ng/ml for PAPP-A.

### Statistical Analysis

To compare analyte concentrations across gestation, we used the one-way ANOVA. To correct for the change across gestation, we expressed all data points as multiples of the normal median (MoMs). We then compared MoMs between miscarriage and controls using the Mann-Whitney-U test. We found PAPP-A levels were undetectable in many normal pregnancies at 5 and 6 weeks gestation, which meant it was not possible to calculate MoMs for participants presenting at these gestations. Therefore, we only compared analyte levels between miscarriage and controls in the cohort at 7-12 weeks gestation where MoMs could be derived.

We then performed multiple logistic regression analysis on the MoMs values to calculate the diagnostic performance of these analytes in predicting miscarriage. We performed receiver-operated characteristics curve analysis to determine the cut-off values for the analytes predict miscarriage. We determined sensitivities, specificities, area under the curve, and positive/negative likelihood ratios of these potential predictive biomarkers. As we were unable to determine MoMs for PAPP-A at 5 and 6 weeks gestation, we did not include PAPP-A in this analysis. Therefore, the diagnostic performance of the following analyte combinations was determined: MIC-1 alone, hCG alone, and MIC-1 with hCG. All analyses were performed using GraphPad Prism (GraphPad Software, La Jolla, CA) except the multiple logistic regression analysis, which was done using Stata^TM^ version 12 (StataCorp LP).

## Results

### Baseline clinical characteristics

We prospectively recruited 462 women presenting to the EPAU where there was ultrasound evidence that the pregnancy was viable (fetal cardiac activity) on the day of presentation. Most of these women presented because they had had symptoms of threatened miscarriage: 40.0% (n=185) presented with vaginal bleeding, 20.3% (n=94) presented with both vaginal bleeding and pain, 19.3% (n=89%) presented with pelvic pain and 20.3% (n=94) did not present with symptoms.

7.4% (n=34) subsequently miscarried and the remaining 428 had ongoing pregnancies and livebirths (controls). The baseline clinical characteristics, stratified to according whether they miscarried or had an ongoing pregnancy, are shown in table 1. The two groups were relatively comparable, except those who miscarried were more likely to present at an earlier gestation. There were no differences in maternal age, body mass index, number of previous miscarriages or parity.

### Plasma MIC-1 concentrations

**Figure 1:**
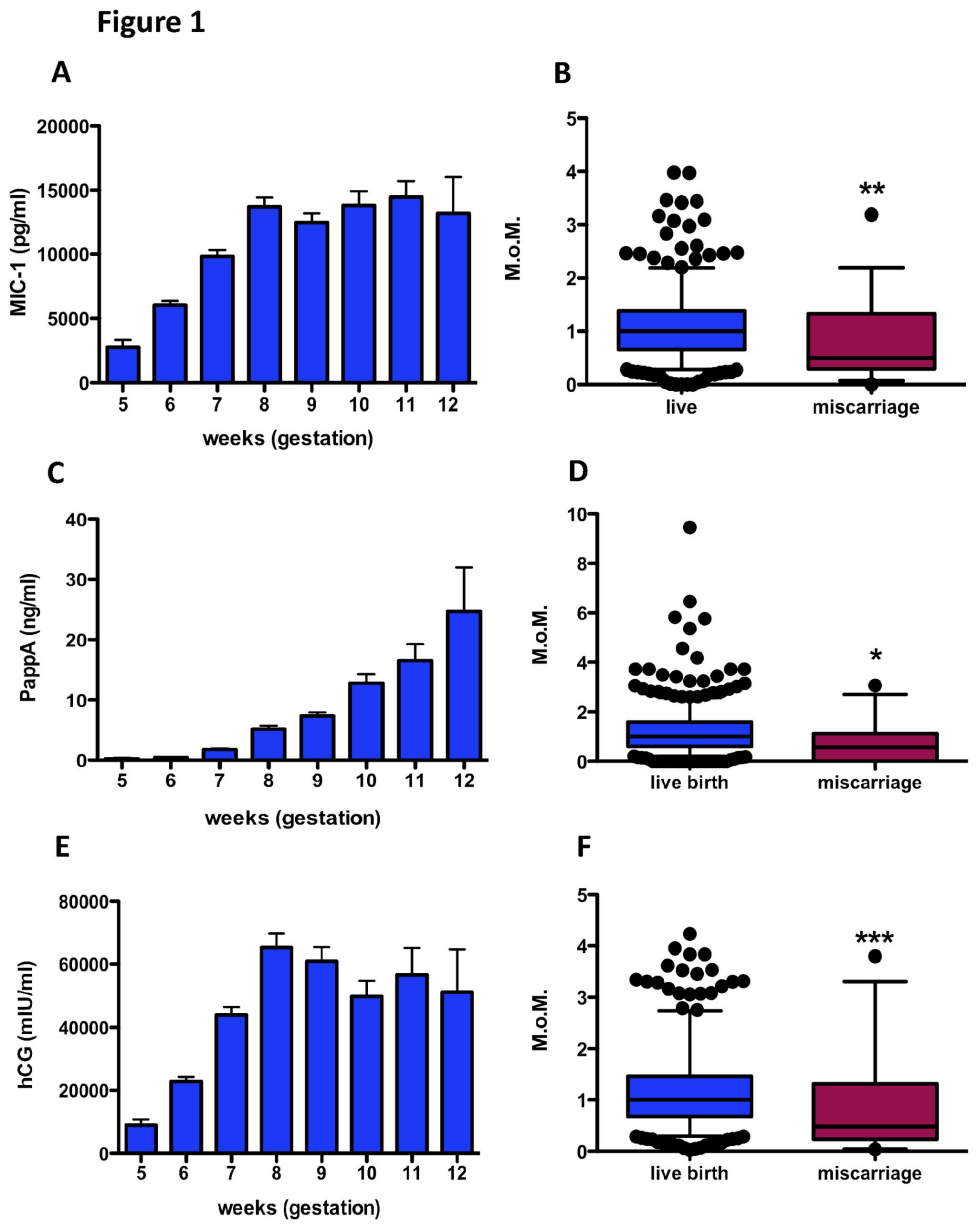
Plasma MIC-1, PAPP-A and hCG concentrations. (A) Mean MIC-1 levels across gestation. (B) Box and Whisker plots of MoM MIC1 levels in the ongoing pregnancy (n=428) and miscarriage cohorts (n=34) (C) Mean PAPP-A levels across gestation (D) Box and Whisker plots MoM PAPP-A levels in the ongoing pregnancy (n=312) and miscarriage cohort (n=11) (E) Mean hCG levels across gestation (F) Box and Whisker plots of MoM hCG levels in the ongoing pregnancy (n=428) and miscarriage (n=34) cohorts. *P=0.036, **P=0.0025, *** P=0.0008. For Figure 1A,C,E, the numbers for each gestation were: 5 weeks n=19; 6 weeks n=132; 7 weeks n=95; 8 weeks n=65; 9 weeks n=54; 10 weeks n=35; 11 weeks n=21; 12 weeks n=6. For Figure 1A,C,E error bars are standard error of the mean.

Plasma MIC-1 levels in the ongoing pregnancy cohort (ie controls) rose steeply across gestation approximately 5 fold (p<0.0001) from 5 to 8 weeks before plateauing (Figure 1A).

To compare MIC-1 levels among those who subsequently miscarried and those who had an ongoing pregnancy, we expressed our data as multiples of the normal median (MoMs) to correct for the increase in analyte levels across gestation. Median MIC-1 among those with a threatened miscarriage and subsequently miscarried were 50% of levels seen among those who ultimately delivered a liveborn at term (Miscarriage cohort MoM 0.50 (25^th^-75^th^ centiles: 0.29-1.33) vs controls MoM 1.00 (0.65-1.38); p=0.0025; Figure 1B). Therefore, plasma MIC-1 levels were significantly decreased among those with a threatened miscarriage and a live fetus, but destined to miscarry.

### Plasma PAPP-A concentrations

There was a very steep increase in PAPP-A concentrations (p<0.0001) across gestation among the liveborn cohort, from 5-12 weeks (Figure 1C). Of note, PAPP-A levels were below the limit of detection of the ELISA for many of the samples in the control group at 5 weeks and 6 weeks. In fact, the median at 5 and 6 weeks for controls was 0, meaning it was not possible to calculate MoMs for these gestations. Therefore, only MoMs from 7-12 weeks could be generated. For this reason, we limited our comparison of PAPP-A between miscarriages (n=11) and controls (n=312) to those at 7-12 weeks gestation. Median PAPP-A MoMs (25^th^-75^th^ centiles) was 0.57 (0.00-1.12) among those who subsequently miscarried, which was significantly lower than those who had a livebirth (MoM 1.00 (0.59-1.59); p=0.036; Figure 1D). Thus, at 7-12 weeks gestation, when plasma PAPP-A can be reliably detected, lower levels were significantly associated with subsequent pregnancy loss.

### Plasma hCG concentrations

Maternal plasma hCG concentrations significantly (p<0.0001) increased up until 8 weeks and then plateaued (Figure 1E). Among those who subsequently miscarried, the median MoMs (25^th^-75^th^ centiles) was 0.48 (0.24-1.32), which was significantly lower than those who ended up with a liveborn (MoM 1.00 (0.67-1.4); p=0.0008; Figure 1F).

### Predictive performance of plasma MIC-1 and hCG concentrations

We then examined the ability of plasma MIC-1 and hCG both alone, and in combination to predict subsequent miscarriage in our cohort. We were unable to include PAPP-A in this analysis since we could not generate MoMs for gestational weeks 5 and 6 (see above).

The predictive performance of MIC-1 was extremely modest. At 90% specificity, the sensitivity was only 9.8%, with an area under the curve of 0.66 (95% CI 0.54-0.78; table 2). hCG alone was also modest with similar diagnostic performance characteristics: at 90% specificity, the sensitivity was 10.9%. Combining both analytes did not improve on the performance of either.

**Table 2:** Diagnostic characteristics of plasma MIC-1 and hCG in predicting pregnancy loss after presenting to an Early Pregnancy Assessment Unit.

**Specificity**	**Sensitivity**	**% correctly identified**	**+ ve likelihood ratio**	**- ve likelihood ratio**	**Area under the curve [95% confidence interval**]
**MIC-1 (MoM**)					**0.66 [0.54-0.78]**
85%	15%	20.1%	1.01	0.99	
90%	9.8%	15.6%	0.98	1.00	
95%	8.6%	15.0%	1.72	0.96	
100%	1.2%	8.4%	-	0.99	
**HCG (MoM**)					**0.67 [0.55-0.79]**
85%	14.0%	19.3%	0.93	1.01	
90%	10.9%	16.7%	1.1	0.99	
95%	3.5%	10.2%	0.70	1.02	
100%	0.9%	8.2%	-	0.99	
**MIC1 and hCG (MoMs**)** combined**					**0.67 [0.54-0.80]**
85%	14.7%	19.9%	0.98	1.00	
90%	8.6%	14.7%	0.86	1.02	
95%	4.2%	10.8%	0.84	1.01	
100%	3.7%	10.8%	-	0.96	

## Discussion

Previously, we had shown in a prospective study of an asymptomatic cohort in early pregnancy that MIC-1 and PAPP-A may have promise as predictive biomarkers for subsequent miscarriage [10]. To further examine this possibility, we undertook this present prospective study in a clinical relevant setting: women presenting to EPAU. Most were referred because they had symptoms of threatened miscarriage (80%), meaning this population was different to the asymptomatic cohort examined in the previous study. In this study, we again confirmed low MIC-1 and PAPP-A concentrations in women with live fetuses are significantly associated with subsequent miscarriage.

A number of groups have sought to identify a predictive biomarker that can be performed in early pregnancy to identify those with live fetuses whom will subsequently miscarry. However, an accurate, validated and predictive biomarker reaching the benchmark of clinical utility has yet to be described. We have previously reported serum Anandamide may be predictive in the situation of threatened miscarriage, although these findings require verification [11]. Muttukrishna et al. reported that among a cohort of 40 women who had a threatened miscarriage, sFlt-1 were significantly depressed among those who miscarried [12]. However, the numbers in that cohort were modest, and no data on the performance of the test was presented (sensitivity/specificity) meaning their findings also require verification. In a prospective study of 122 participants, Johns et al. concluded inhibin A was decreased among those who subsequently aborted, but the area under the receiver operating characteristic curve was somewhat modest (0.68 when the results were combined with hCG) [13].

While MIC-1 levels were significantly decreased among those destined to miscarry, we were disappointed to find that it performs very modestly as a predictive biomarker test for miscarriage in this largely symptomatic cohort. Interestingly, the ability of MIC-1 to predict miscarriage appears substantially better when applied to an asymptomatic cohort (57% sensitivity at 90% specificity as a lone marker) [10] compared to the present population presenting to the EPAU where a majority had symptoms of threatened miscarriage (9.8% sensitivity at 90% specificity to predict miscarriage). There are a number of possible explanations for this rather surprising difference in our two independent prospective studies. First, in the asymptomatic cohort [10] we used serum whereas plasma was collected in the present study. Secondly, it is possible that MIC-1 may be genuinely more reliably depressed at an earlier stage of an evolving miscarriage when women are still asymptomatic, rather than later in the pathophysiological process when clinical signs have become apparent (when significant perigestational haemorrhage may have occurred).

Owing to the fact PAPP-A was not detectable in a substantial number of samples at 5 and 6 weeks gestation, we were unable to generate MoMs for these cohorts. As such, we were unable to evaluate the diagnostic performance of PAPP-A in our cohort. The fact that plasma levels of PAPP-A are apparently not detectable in a significant proportion of normal pregnancies at 5-6 weeks suggests it would seem unlikely plasma PAPP-A could be relied upon as a clinical biomarker for miscarriage.

We believe our study has some strengths. It was a prospective study of significant numbers, specifically designed to identify predictive markers of miscarriage. Only one investigator (K.B.) recruited all participants, collected and processed all samples. Furthermore, we only recruited women with a viable pregnancy (fetal cardiac activity) at the time of blood sampling. Finally, the assays were done in batch, by an investigator blinded to clinical outcomes.

While plasma levels might not be useful as a predictive test, our data strongly supports the association between low MIC-1 [1,10], PAPP-A [9,10] in evolving pregnancy loss. This premise is supported by our prospective study in asymptomatic women, which arrived at the same conclusions [10].

Given levels are low in evolving pregnancy loss, it is likely that MIC-1 and PAPP-A play important biological roles in the maintenance of early pregnancy. In support of this contention is that their known biological functions are consistent with that of pregnancy maintenance. Recent data suggests MIC-1 promotes an increase in a tolerogenic subtype of dendritic cells in the decidua [8]. PAPP-A is a protease of insulin-growth factor binding protein-4 [14]. By cleaving insulin-growth factor binding protein 4, PAPP-A may be increasing free and bioactive insulin-like growth factors I and II. There is functional in vitro data suggesting these IGFs may have roles in promoting healthy placentation [15-18]. As such, we strongly contend these proteins merit attention and deserve further interrogation of their biological function in early pregnancy. This is especially true for MIC-1, which has been relatively poorly studied in early pregnancy.

In conclusion, we have confirmed plasma MIC-1, PAPP-A and hCG concentrations are depressed in women presenting to EPAU with a viable embryo, but later miscarry. While they do not appear to perform well as predictive biomarkers in this clinical setting, they are likely to play an important role in both healthy pregnancies and possibly in evolving pregnancy failure.
